# Harnessing Biopolymer Gels for Theranostic Applications: Imaging Agent Integration and Real-Time Monitoring of Drug Delivery

**DOI:** 10.3390/gels10080535

**Published:** 2024-08-14

**Authors:** Pranita Jirvankar, Surendra Agrawal, Nikhita Chambhare, Rishabh Agrawal

**Affiliations:** 1 Datta Meghe College of Pharmacy, Datta Meghe Institute of Higher Education and Research (Deemed to Be University), Wardha 442001, Maharashtra, India; pjirvankar123@gmail.com (P.J.); chambharenikhita@gmail.com (N.C.); 2 Bajiraoji Karanjekar College of Pharmacy, Sakoli 441802, Maharashtra, India; agrawalrishabh142@gmail.com

**Keywords:** biopolymer gel, real-time monitoring, imaging agent

## Abstract

Biopolymer gels have gained tremendous potential for therapeutic applications due to their biocompatibility, biodegradability, and ability to adsorb and bind biological fluids, making them attractive for drug delivery and therapy. In this study, the versatility of biopolymer gels is explored in theranostic backgrounds, with a focus on integrating imaging features and facilitating real-time monitoring of drug delivery. Different methods of delivery are explored for incorporating imaging agents into biopolymer gels, including encapsulation, surface functionalization, nanoparticle encapsulation, and layer-by-layer assembly techniques. These methods exhibit the integration of agents and real-time monitoring drug delivery. We summarize the synthesis methods, general properties, and functional mechanisms of biopolymer gels, demonstrating their broad applications as multimodal systems for imaging-based therapeutics. These techniques not only enable multiple imaging but also provide signal enhancement and facilitate imaging targets, increasing the diagnostic accuracy and therapeutic efficacy. In addition, current techniques for incorporating imaging agents into biopolymer gels are discussed, as well as their role in precise drug delivery and monitoring.

## 1. Introduction

Biopolymer gels have gained significant attention in recent years for their potential use in drug delivery platforms and theranostic applications. Biocompatibility and biodegradability are fundamental necessities for biomedical applications, and biopolymeric hydrogels are especially valuable in the organization of medications and theranostics because of their capacity to retain and hold a significant measure of water and natural media [1]. Theranostic viability can be expanded by reorganizing drug loading and controlling delivery attributes under specific biological feeling conditions as per the exceptional permeable organization construction of hydrogels in light of biopolymers [1]. Since biopolymers arrive in an extensive variety of microgel/nanogel structures that are great for and harmless to the vast majority of ecosystem applications, they are believed to be a decent choice for tending to biocompatibility and biodegradability challenges [2]. The capability of biopolymer-based microgels/nanogels as medication transporters in biomedical applications has been researched; studies have featured the utilization of a few biopolymers in the plan of nanogels and flow improvements in drug and biomolecule conveyance applications. The utilization of microgels/nanogels as medication transporters and their development using regular gums and their derivatives are of immense importance [3].

**Figure 1:**
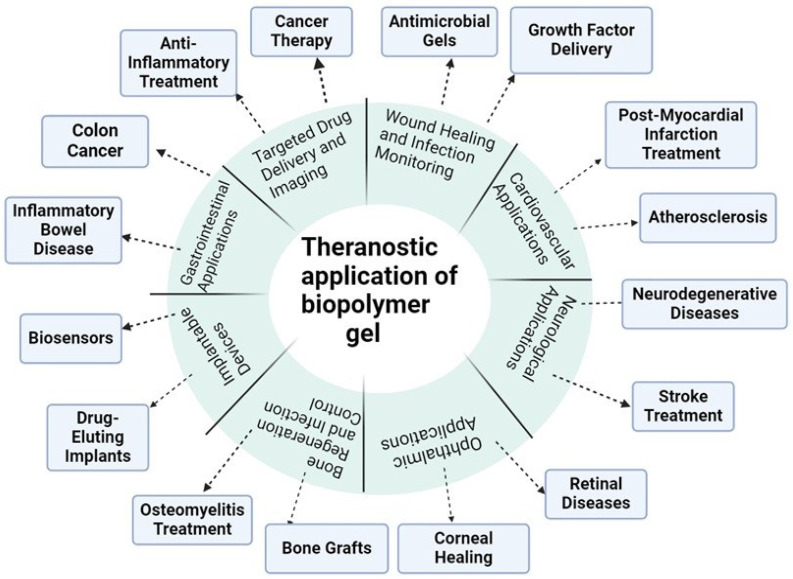
Theranostic applications of the biopolymer gel.

Biopolymer-based nanogels have additionally been researched for their capability to ship medicines and biomolecules because of their high retention limit of water and natural liquids [4]. Surveys have examined the utilization of different biopolymer-based microgels or nanogels for drug conveyance in biomedical applications, as listed in Figure 1. They underline the headway made in epitomizing atoms with different restorative purposes utilizing biopolymer-based nano/microgels, as well as clever ways to deal with amalgamation, or potentially functionalization, to upgrade their conveyance capacities. In the current field of medication, the rising significance of the union among diagnostics and treatments has prompted the development of the theranostic worldview [5]. This coordinated methodology is focused on effectively connecting indicative capacities with restorative activities, empowering the arrangement of individualized and exact treatments for different problems. A huge commitment is held by theranostic procedures for working on understanding results while limiting secondary effects, as they give continuous information on treatment adequacy and illness movement [6].

As the field of medication goes through a worldview change, theranostics rises above conventional limits for determination and treatment. Utilizing a synergistic methodology that consolidates restorative methodologies coordinated by demonstrative strategies, it gives exact and tweaked patient consideration. Through early ailment ID and the constant observation of treatment reaction, doctors might fit treatment techniques to every patient’s biopolymer gels, which certainly stand out lately for their possible use in drug conveyance stages and theranostic applications [7]. Biocompatibility and biodegradability are necessities for biomedical applications, and biopolymeric hydrogels are especially helpful in the organization of medications and theranostics because of their capacity to assimilate and hold a significant measure of water and natural media [8]. Theranostic viability can be expanded by advancing medication stacking and controlling delivery qualities under specific organic excitement conditions, as indicated by the exceptional permeable organization design of hydrogels in view of biopolymers [9]. Since biopolymers arrive in an extensive variety of microgel/nanogel structures that are great for and harmless to the majority of the ecosystem applications, they are believed to be a decent choice for tending to biocompatibility and biodegradability challenges [10]. The capability of biopolymer-based microgels/nanogels as medication transporters in biomedical applications has been examined; studies have featured the utilization of a few biopolymers in the plan of nanogels and their ongoing improvements in drug and biomolecule conveyance applications. The utilization of micro/nanogels as medication transporters and the creation processes using regular gums and their subordinates are additionally remembered for the surveys [11].

Biopolymer-based nanogels have additionally been explored for their capability to move drugs and biomolecules because of their high ingestion limit of water and natural liquids [12]. The authors examine the utilization of different biopolymer-based microgels and nanogels for drug conveyance in biomedical applications. They underline the headway made in epitomizing particles with different restorative purposes utilizing biopolymer-based nano/microgels, as well as clever ways to deal with combination or potentially functionalization to improve their conveyance abilities [13]. In present-day medication, the rising significance of the assembly among diagnostics and treatments has prompted the development of the theranostic worldview. This coordinated methodology is focused on effectively connecting demonstrative abilities with restorative activities, empowering the arrangement of individualized and exact treatments for different problems. Tremendous commitment is held by theranostic strategies for working on quiet results while limiting aftereffects, as they give constant information on treatment viability and sickness movement [14].

Finally, theranostics as a worldview change in the field of medication transcends the conventional limits for determination and treatment. It is anything but a synergistic methodology combining the remedial methodologies coordinated by demonstrative strategies for giving exact and customized patient consideration. Theranostics provides distinguishing proof for early sickness and tries to monitor treatment reaction so many treatment methodologies can be adjusted to patients’ particular necessities. Besides, theranostic approaches may improve helpful outcomes by limiting adverse consequences and improving medication circulation. As a result of their excellent features, including biocompatibility, mechanical qualities that may be readily functionalized, and ease of use, theranostic applications have discovered excellent flexibility in biopolymer gels [15]. These gels, which are created with highly regular, synthetic polymers, can be utilized to create a three-layered network structure capable of holding medical substances and imaging probes. The important point is that drug delivery might be prompted by certain physiological signs or external factors through the fabrication of a bio-polymer gel that exhibits a responsive stimulus. As well, compared to traditional drug delivery systems, their organic biodegradability can significantly reduce the problems of toxicity and accumulating for a long time [16].

Biopolymer gels such as alginate, gelatin, and chitosan are used to create scaffolds that support cell growth and tissue formation. Their biocompatibility and biodegradability are crucial for successful tissue regeneration. Moreover, 3D-printed biopolymer gels can be designed to deliver drugs precisely at the site of interest. For example, hydrogels made from alginate or gelatin can be loaded with chemotherapy drugs and printed into shapes that fit the tumor site exactly. Biopolymer gels can encapsulate living cells, allowing for the creation of complex tissue structures [17]. Hydrogels like collagen and hyaluronic acid provide a supportive environment for cell proliferation and differentiation. Biopolymer gels can be combined with other materials, such as nanoparticles or fibers, to enhance their properties for specific applications. Biopolymer gels-based implants not only deliver drugs but also monitor therapeutic outcomes. For example, a 3D-printed implant releases anti-inflammatory drugs while simultaneously measuring local inflammation levels [18].

**Figure 2:**
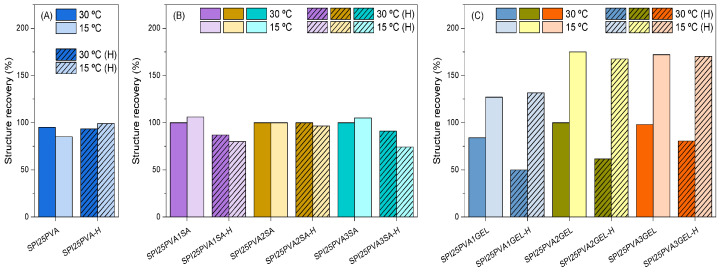
Structure recovery percentage for (**A**) SPI25PVA inks with (**B**) different contents of SA (1–3 wt%) and (**C**) different contents of GEL (1–3 wt%) as a function of test temperature (dark color, 30 °C; light color, 15 °C). Line patterns are used for heat-treated samples (designated with an H at the end of the name).

In order to assess protein–polysaccharide and protein–protein interactions, adjustments were made to soybean protein isolate (SPI) inks based on heat treatment and biopolymer concentration. Initially, the inks were analyzed to determine their rheological properties in relation to the concentration of polyvinyl alcohol (PVA) used (20, 25, and 30 wt%). After careful evaluation, the ink with a PVA concentration of 25 wt% was selected. For the purpose of enhancing ink viscoelasticity, the formulations were supplemented with sodium alginate (SA) and gelatin (GEL). The impact of different concentrations of SA or GEL (1–3 wt%) was then investigated. This test simulates platform 3D printing ink deposition. Figure 2A shows the recovery percentage of 25 wt% PVA samples, where test temperature (15 or 30 °C) and ink heat treatment had no significant effect. Figure 2B shows that alginate inks recovered similarly regardless of test temperature, but heat-treated inks recovered slightly less. Inks with varying GEL compositions (Figure 2C) showed different recovery percentages at different test temperatures, with larger recovery percentages at 15 °C. Gelatin’s heat reversibility in generating intramolecular hydrogen bonds causes gelation [19].

Theranostic approaches can also help to improve the outcomes of restorative therapy through diminishing unwanted effects and improving drug delivery. These methods have displayed an exceptional flexibility in biopolymer gels due to their peculiarities such as biocompatibility, easily modifiable mechanical characteristics, and user-friendliness. These natural or artificial polymer gels can be organized into three-layer network structure capable of retaining therapeutic substances and imaging agents. However, it is important to note that bio-polymeric gels are stimuli-responsive and so can deliver drugs upon particular physiological signals or external stimuli [20].

## 2. Intersecting Diagnosis with Therapy

The word “theranostics”, which combines the words “therapy” and “diagnostics”, signifies a concept that emphasizes integrating diagnostic capabilities with therapeutic treatments. In most cases, traditional means of diagnosis are used prior to making treatment choices; hence, there is a systematic approach to patient care with a degree of fragmentation involved. Conversely, theranostics fills this void by delivering therapeutic agents alongside providing timely assessment of treatment efficiency [21]. This integration provides broad knowledge about the progression of illnesses and outcome of treatments, thus making it easy for a clinician to interact with patients. This is because theranostic platforms are able to detect an infection at early stages, follow the progress made in treatment, and adjust treatment schedules. The use of diagnostic techniques such as imaging or biomarker analysis enables health care practitioners to understand the nature of a disease in terms of its features, molecular markers, and spatial distribution of pathologic lesions. These details thereby assist in selecting and planning treatment procedures that are rational, timely, specific, and individualized per the patient’s requirements. Furthermore, due to continuous interaction between therapies and diagnostics, there is flexibility when choosing various approaches to therapy that lead to decreased side effects and development of resistance therapies [22].

## 3. Biopolymer Gels as Versatile Platforms

Biopolymer gels have been described as biocompatible, tunable, and they can be functionalized easily, making them ideal for a variety of biomedical applications. The positive features of these materials are due to the fact that they create a three-dimensional structure having high water content, which is similar to the living tissues’ extracellular matrix environment through natural and synthetic polymers. As such, they exhibit improved cellular interactions, adjustable mechanical properties, sustained drug release kinetics, and other advantageous traits that resemble those of naturally occurring biological systems [23].

The responsive gelation process is one reason among many others why biopolymer gels are highly versatile. When exposed to environmental conditions like changes in pH, temperature variations, light, etc., the gelling state may change reversibly. In effecting controlled release drug delivery this system has precise control over drug release kinetics [24].

**Figure 3:**
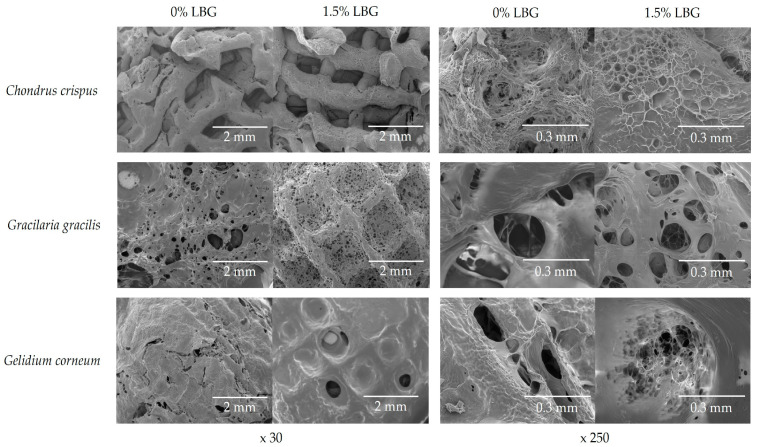
SEM images of *C. crispus*, *G. gracilis*, and *G. Corneum* 3D gels, with and without LBG addition, under 30× and 250× magnifications.

Local companies used *Chandrus crispus*, *Gelidium Corneum,* and *Gracilaria gracilis* as culinary additives due to their gelling biopolymers. Hydration at room temperature, stirring at 90 °C, and centrifugation at 40 °C helped extract these biopolymers. These extracts were tested as bioinks for extrusion-based 3D printers. The shape, size, and number of holes in the gel provide insights into the molecular interactions within seaweed gels, both with and without LBG. When the concentration of LBG was increased to 1.5%, the resulting gels exhibited a higher density and smaller pore size. In particular, the gels derived from *C. crispus* and *G. gelidium* exhibited a stronger synergistic interaction between the κ/ι-carrageenan and agar components. On the other hand, *G. gracilis* exhibited broader openings and a less compact microstructure, as shown in Figure 3. The variety of seaweed gels was quite extensive. Past studies have indicated that mixed system gels exhibit a higher level of heterogeneity compared to gels made from individual gelling components [25].

Furthermore, biopolymer gels can be designed specifically for certain types of cells or molecules; reducing off-target effects therefore enables targeted treatment. Biopolymer gels are used because of their porous nature that allows the incorporation of imaging agents. This means images can be captured and treatment response monitored in real time. Biopolymer-based theranostic platforms can be considered as heterogeneous with no restrictions between diagnosis and treatment due to the direct incorporation of imaging agents into the gel structure, thus opening the door to individualized therapeutic approaches [23].

To accomplish localized cancer therapy and tumor imaging, injectable hydrogels preloaded with chemotherapeutic medications and imaging contrast agents can be utilized. Polyethylene glycol (PEG) and chitosan can be coupled with the contrast agent gadolinium and the chemotherapeutic drug doxorubicin. The hydrogel can target the tumor site precisely after injection, making it possible to continuously release medication and make tumor MRI imaging easier. Growth hormones, such as epidermal growth factor (EGF), and pH sensors can be included into hydrogels composed of gelatin and hyaluronic acid. When applied to wounds, this hydrogel can hasten the healing process and provide instantaneous information about the surrounding environment, assisting in the proper course of therapy [26].

Nanogels have been fitted with MRI contrast agents and altered to target cancer cells with ligands. Folic acid conjugation with poly(lactic-co-glycolic acid) (PLGA) and PNIPAM nanogels allows for the selective targeting of cancer cells expressing overexpressed folate receptors [27]. The nanogels have the capacity to transfer superparamagnetic iron oxide nanoparticles (SPIONs) for magnetic resonance imaging, together with the chemotherapeutic drug paclitaxel. This makes it possible to view cancers and treat them specifically. It is possible to incorporate a fluorescent marker and plasmid DNA containing a therapeutic gene into chitosan-based nanogels. The gene is triggered in the target cells upon delivery, and the fluorescent marker aids in monitoring the location and efficacy of gene expression [28].

The PEG and alginate cryogel can incorporate glucose oxidase and doxorubicin sensors. This scaffold can be placed in the location where a tumor was excised in order to track blood sugar levels and administer medication there. This makes it possible to evaluate the tumor’s response to therapy as well as its metabolism [29]. Collagen and hyaluronic acid cryogels have the ability to integrate growth agents and oxygen sensors. Cryogels have the capacity to replenish bone or cartilage and provide instantaneous oxygen level readings. For tissues to operate and survive as best they can, this knowledge is essential [30].

## 4. Properties and Characteristics of Biopolymer Gels

**Figure 4:**
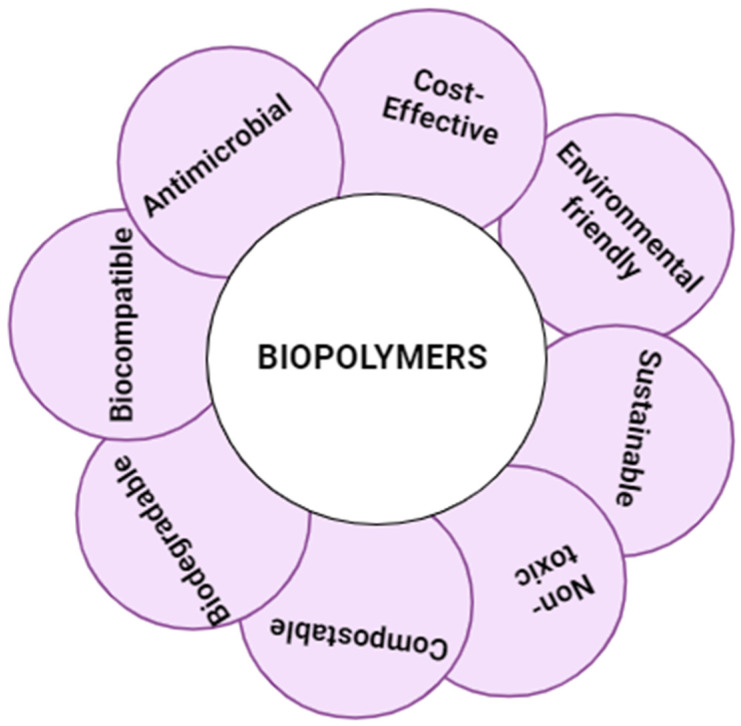
Properties of biopolymer gels.

Biopolymer gels are multifunctional materials with unique properties that make them ideal for various biomedical applications such as drug delivery, tissue engineering, theranostics, etc. These gels contain natural or synthetic polymers that can form crosslinked networks, so they offer a variety of options [31]. Natural polymers such as alginate, chitosan, collagen, hyaluronic acid, and gelatin have inherent biocompatibility while their man-made counterparts such as polyethylene glycol (PEG), polyvinyl alcohol (PVA), and poly(N-isopropylacrylamide) also offer adjustability for features such as improved mechanical strength [32]. Some important properties affecting the three-dimensional interface structure of biopolymer gels are mechanical properties, pore size distribution, and drug release kinetics. Manipulation of parameters such as crosslinking density and polymer concentration can be a variety of factors. These gels respond to external stimuli such as temperature, pH, or light and therefore can be reversibly modified [33]. The interaction between negatively charged alginate and positively charged chitosan results in the formation of a stable gel with favorable mechanical properties. The gel has the capacity to incorporate and retain a significant amount of water due to the hydrophilic nature of both PVA and PAA. The gel’s characteristics can be modified by modifying the ionic content, which is advantageous for sensor applications [34]. The mechanical characteristics of gelatin–polyethylene glycol can be altered by adjusting the gelatin to polyethylene glycol ratios. The thermosensitive properties of these polymers enable them to solidify at body temperature. It is possible to engineer gels composed of hyaluronic acid and poly(lactic-co-glycolic acid) (PLGA) to discharge medications or other bioactive substances under controlled conditions. PLGA imparts mechanical strength to the hyaluronic acid gel, which is otherwise pliable. The mechanical resilience of agarose-poly(N-isopropylacrylamide) (PNIPAAm) gels enables reversible sol–gel transitions. The lower critical solution temperature (LCST) of the well-known thermoresponsive polymer poly(N-isopropylacrylamide) (PNIPAM) is approximately 32 °C. The flexibility of PNIPAM-based gels and polymers is advantageous for wearable and conformable applications. The PNIPAM–chitosan gel exhibits dual stimuli responsiveness by reacting to changes in temperature and pH [35]. These characteristics make them suitable for targeting as their mechanism of action enhances their drug delivery to be useful in a variety of applications. Biodegradability and biocompatibility are important considerations when organisms in biopolymers mimic aspects of the extracellular matrix that support cell adhesion and tissue regeneration [36]. Controlled cell degradation is important for gel breakdown into non-toxic products, which is key to maintaining long-term drug release and reducing side effects, as listed in Figure 4. Furthermore, the scalable mechanism of biopolymer gels is important because it can mimic native tissue and withstand physiological stress. This flexibility makes them useful for a variety of biomedical applications, including clinical practice and diagnostics [37].

## 5. Imaging Agents in Theranostic Applications

Imaging modalities play an important role in treatment by providing valuable information on disease location, progression, and treatment response as listed in Table 1 [38].

Imaging techniques covered several techniques based on physical principles, such as X-ray, ultrasound, magnetic resonance, fluorescence, and nuclear imaging, and these techniques produce detailed images with high-quality spatial and temporal resolution. Each modality has specific advantages and limitations, making it suitable for different applications in theranostics [39].

X-ray imaging such as radiography and computed tomography (CT) relies on the contrast of X-rays absorbed by tissues to create detailed images of the body. This technique is commonly used to detect bone fractures, and diagnose and monitor tumors, which determine how doctors intervene [40].

Ultrasound imaging uses high-frequency sound waves to create real-time images of organs and tissues in the body. This noninvasive, portable, and radiation-free technique is suitable for a variety of diagnostic and interventional procedures [41].

MRI technology relies on magnetic fields and radio waves to generate contrast images of soft tissue, ideally suited to imaging the brain, spinal cord, and musculoskeletal systems, as well as detect tumors and inflammation [42].

Fluorescence imaging uses fluorescent dyes or materials that emit light upon excitation by an external source, enabling the detection of specific molecular targets, biomarkers, or cellular processes with high sensitivity and specificity [43].

Nuclear imaging modalities, such as positron emission tomography (PET) and single-photon emission computed tomography (SPECT), use radioactivity for detailed functional imaging to provide quantitative information on tracer uptake, metabolism, and biological distribution important for molecular analysis and anatomical imaging [44].

Among the therapies, the selection of appropriate imaging agents is important for accurate diagnosis, treatment planning, and monitoring of therapeutic response. When different imaging modalities and agents are used, individual exposure intervention can benefit from improved outcomes [45].

**Table 1:** Imaging agents and their applications.

Imaging Modality	Imaging Agent	Theranostic Application	
X-ray Imaging	Iodine-based contrast agents	Diagnosis of bone fractures, detection of tumors, and monitoring therapeutic interventions	[46]
	Barium sulfate	Visualization of gastrointestinal tract for diagnosing conditions like ulcers or tumors	
Ultrasound Imaging	Microbubble contrast agents	Assessing blood flow, visualizing organs, and guiding interventional procedures	[47]
	Contrast-enhanced ultrasound	Imaging liver lesions, assessing vascularity in tumors, and diagnosing cardiovascular conditions	
Magnetic Resonance Imaging	Gadolinium-based contrast agents	Imaging brain, spinal cord, and musculoskeletal system, and detecting tumors and inflammatory processes	[48]
	Superparamagnetic iron oxide nanoparticles	Targeted drug delivery and imaging of inflammation	
Fluorescence Imaging	Fluorescent dyes	Visualizing specific molecular targets, biomarkers, or cellular processes with high sensitivity	[49]
	Quantum dots	Multiplexed imaging of molecular targets for personalized medicine	
Nuclear Imaging	Fluorodeoxyglucose	Cancer diagnosis and monitoring response to treatment	[50]
	Technetium-99m labeled agents	Imaging myocardial perfusion and diagnosing bone metastases	
	Copper-64 labeled nanoparticles	Imaging and tracking of stem cell therapy	

## 6. Importance of Imaging Agents in Theranostics

In theranostics, dual roles are served by imaging agents as diagnostic tools and therapeutic agents, enabling the integration of diagnostics and therapeutics into a unified platform. Through the incorporation of imaging agents directly into therapeutic formulations or delivery vehicles, disease progression can be monitored, treatment response assessed, and therapeutic regimens optimized in real time by clinicians [51]. Non-invasive visualization of drug distribution, pharmacokinetics, and pharmacodynamics is facilitated by imaging agents, allowing for personalized treatment strategies tailored to individual patient profiles. Moreover, early detection of treatment resistance, identification of therapeutic targets, and prediction of treatment outcomes are enabled by imaging agents, thereby improving patient outcomes and reducing healthcare costs [52].

**Figure 5:**
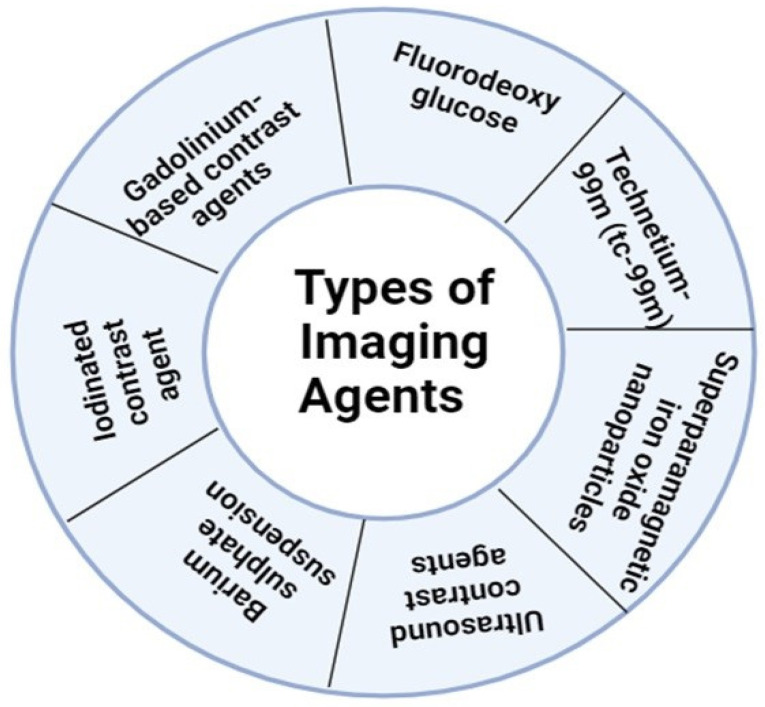
Types of imaging agents.

Numerous imaging agents are available for use in theranostic applications, such as contrast agents, fluorescent dyes and probes, radiotracers, nanoparticles, and molecular probes, each possessing unique properties and applications, as listed in Figure 5.

Contrast agents enhance the visibility of anatomical structures or pathological lesions on imaging scans by altering the contrast between tissues. Examples include iodinated contrast agents for X-ray and CT imaging, gadolinium-based contrast agents for MRI, and microbubbles for ultrasound imaging. Fluorescent dyes and probes emit light at specific wavelengths upon excitation by external sources, enabling visualization of molecular targets, cellular processes, and biological interactions [53]. They are commonly used in preclinical research and intraoperative imaging for guiding surgical resection of tumors. Nanoparticles that contain imaging agents such as quantum dots, iron oxide nanoparticles, and gold nanoparticles make possible multimodal imaging and focused drug delivery. They are important as they have more stability, a long circulation time, and can be targeted towards specific sites. Molecular probes have been developed to target disease-specific biomarkers or molecular pathways, thus allowing selective imaging of different diseases. Consequently, they can be tagged with fluorescent dyes, radioactive tracers, or MRI contrast agents for substantial results through several imaging methods [54].

## 7. Integration of Imaging Agents with Biopolymer Gels

Combining biopolymer gels with diagnostic tools improves diagnosis, therapy, and monitoring. Signal strength is improved through strategies which enhance biocompatibility with respect to biomedical applications in safety terms.

Different methods have been developed for incorporating imaging agents inside biopolymer gels, allowing multimodal imaging and monitoring therapeutic interventions in real time [55].

Encapsulation: During gelation, the imaging agents can be enclosed inside the gel matrix via physical entrapment or chemical conjugation. For example, prior to the gel formation, fluorescent dyes, contrast agents, or radiotracers can be mixed with polymer solution to ensure that they are uniformly distributed within the gel matrix [56].

Surface functionalization of biopolymer gels can be modified by targeting ligands or affinity moieties to specifically bind a range of imaging agents to enhance their retention and localization in the supervised area [57].

The versatility and potential of biopolymer-based imaging agents for theranostic applications, including their ability to be tailored for specific biomedical applications, has been illustrated in Table 2. Their biocompatibility and biodegradability, and their potential for real-time monitoring of treatment responses, are witnessed by these examples.

## 8. Role of Biopolymer Gels in Real-Time Monitoring

A few strategies can be employed in combination with biopolymer gels to improve imaging signals and increase the sensitivity and specificity of imaging modalities.

Combining biopolymer gels with imaging techniques offers a promising approach to improve sensitivity and specificity in medical imaging. Signal enhancement techniques, such as conjugation of signal amplification probes and imaging agents of nanoparticles or enzymes, enable the detection of low-level imaging materials with high sensitivity and resolution. Furthermore, many images using biopolymer gels with imaging techniques are used to obtain and provide coverage information, compensate for individual errors, and even provide accurate analysis, selective imaging obtained by targeting biopolymer gels with ligands or antibodies [66]. An operationalization facilitates accurate localization of infectious lesions and early diagnosis, thereby guiding treatment decisions for better patient outcomes [67].

In theranostic platforms, biocompatibility considerations are most important. In vitro cytotoxicity assessment provides important information about the compatibility of imaging agents and biopolymer gels with cells. The optimization of medication loaded in the biopolymer gels to ensure a safe in vivo experience by surface modification methods, and the degradation of biopolymer gels by phototherapy treatment representative for a period of time, is crucial. Beneficial substances are released sequentially, where the deleterious substances are non-toxic and are easily metabolized or eliminated from the body. Compliance with regulatory requirements including clinical safety assessment is essential for developing treatment to ensure individual safety and efficiency [68].

Biopolymer gels have several compelling features to be employed in real-time tracking of drug administration. First, the crucial advantage is their tunability and stimuli-responsive behavior, which allows their controlled dissolution and disintegration upon external action. They can also readily encapsulate imaging agents within their matrix, which permits monitoring of the release kinetics or in situ distribution within the target area. The use of biopolymer gels provides for on-demand changes in the drug release rate based on physiological conditions or external agents, which also significantly increases treatment selectivity. Moreover, biopolymer gels simultaneously act as reservoirs for sustained release of the drug and carriers for its delivery. Biopolymer gels can further be engineered to possess specific properties, such as injectability, shear-thinning behavior, or mucoadhesive properties to allow for facile minimally invasive administration and localized drug delivery to disease tissue. This allows maximal patient comfort and adherence and minimizes the need for frequent dosing, making them more suitable for chronic or localized diseases [5].

The incorporation of biopolymer gels into real-time monitoring techniques holds immense potential across various fields such as drug delivery, tissue engineering, and regenerative medicine [69]. In targeted cancer therapy, biopolymer-based drug delivery systems enable precise delivery of therapeutic agents to tumor tissues, minimizing systemic toxicity and enabling individualized treatment plans based on real-time monitoring of drug delivery rhythms and tumor response. Moreover, biopolymer gels offer controlled drug release, making them ideal for long-term intervention therapy in chronic diseases like diabetes and heart disease, while also facilitating tissue regeneration and wound healing in regenerative medicine applications [70]. Real-time monitoring of drug release kinetics and tissue remodeling processes enhances therapeutic efficacy and enables customized treatment strategies tailored to individual patient needs.

Recent advancements in biopolymer gel formulations, imaging techniques, and monitoring technologies have propelled the development of next-generation theranostic systems for personalized medicine and healthcare. Hydrogels, nanogels, cryogels, and other emerging gel systems offer versatile platforms for controlled drug delivery, simultaneous imaging, and real-time analytical capabilities [71]. By leveraging the unique properties of these gel systems, researchers can develop innovative pharmaceutical strategies that combine drug delivery, imaging, and real-time monitoring, paving the way for personalized medicine, regenerative medicine, and precision healthcare approaches that optimize therapeutic outcomes for patients [72].

## 9. Challenges in Conventional Drug Delivery Monitoring

Monitoring drug delivery through traditional techniques is primarily based on indirect measurements with reasonable limitations to quantifying fundamental aspects of drug delivery, such as drug concentration in target organs and drug pharmacokinetic activity in a patient’s body. Common drawbacks of conventional methods are the absence of precision, invasive procedures, significant time delay, and poor sensitivity [73].

Spatial resolution with conventional monitoring approaches, such as blood sampling and tissue biopsies, are the most limiting, as they do not provide insight into localized drug distribution or concentration gradients in target tissues. In many cases, invasive procedures such as tissue sampling or catheter placement are required to monitor these techniques. These strategies may result in patient discomfort, the risk of infection, and procedural risks. Furthermore, the timing of the feedback, including drug delivery kinetics, is poor with conventional monitoring approaches. Currently, feedback is frequently obtained long after administration. Therefore, adjusting treatment or optimizing therapeutic work regimens is challenging [74].

## 10. Future Outlook and Recommendations

With continued advancements in materials science, imaging technology, and biomedical engineering, biopolymer gels have a bright future ahead of them in theranostic applications. These advancements could help to realize the potential of biopolymer gels in biocompatibility and stability issues in general, improve imaging resolution and sensitivity, and address clinical interpretation and regulatory issues. Theranostic applications for nanoparticles coated with biopolymers have been investigated; however, further research is needed on long-term toxicity and safety. In addition to demonstrating the pilots for this work, scale-up technological exploration is required. Investigating the application of biopolymeric gels in conjunction with various nano-formulations such as liposomes, micellar drug delivery, and dendrimers in detail is crucial. To demonstrate the theranostic utility of polymeric gels, further research is needed in the field of their integration with wearable sensors.

## 11. Conclusions

Biopolymer gels have become highly adaptable platforms for theranostic applications, combining imaging, real-time monitoring, and drug administration into cohesive systems for personalized and precision treatment. These gels, which can include imaging agents, are biocompatible, have customizable characteristics, and are made of natural or synthetic polymers that crosslink to form networks that resemble the extracellular matrix. Imaging agents are included into biopolymer gels to allow for real-time drug delivery tracking, precise therapeutic control, and treatment result optimization. The development of more advanced biopolymer gel systems with additional functionalities like stimuli responsiveness or self-healing qualities to further improve their therapeutic potential should be the main focus of future research efforts. These systems can be customized to particular biomedical applications.

## Figures and Tables

**Table 2:** Patents filed on biopolymer gels for therapeutic and diagnostic applications.

S. No.	Patent Number	Material Used	Purpose	Remarks	Ref.
1.	US11969438B1	Vegetable oil-derived polyol	Anti-cancer activity	Provides compositions and methods for selectively treating a cancer or tumor utilizing an effective amount of a vegetable oil-derived polyol or hydrogel particles comprising a vegetable oil-derived polyol; it provides a method of targeting and imaging various tumors and/or tumor-associated macrophages	[58]
2.	US20210322462A1	Polyvinyl alcohol (PVA), collagen-chitosan	Wound prevention and/or treatment	The composition is embedded in a hydrogel made of polyvinyl alcohol (PVA), collagen-chitosan, alginates, carbopol gels, and alginate matrices for slow release	[59]
3.	US10201622B2	Gelatin, casein, dextran, PEG, PVP	Theranostic applications	Nanoparticles coated with polymers like gelatin, casein, dextran, PEG, and PVP addressed the theranostic applications	[60]
4.	US11529430B2	Chitosan, polylactic acid, polyglycolic acid, and copolymers	Contrast agent	Gadolinium DOTA nanoparticles decorated with polydopamine and as a photothermal agent to kill cancer cells	[61]
5.	US9095629B2	PEG and nitro-DOPA	Magnetic nanoparticles	As contrast agent and for better targeting of the MNPs	[62]
6.	US8372944B1	Hyperbranched polyester and hyperbranched polyester amine	Polymeric nanoparticles as theranostic agent	Polymeric nanoparticles coated with HBPE for fluoroscence and delivery of therapeutic drug	[63]
7.	US10799604B2	Polyethylene glycol, polyacrylic acid, polyacrylamide, poly(N-isopropylacrylamide), hyaluronic acid, and combinations thereof	Implant for tumor cell tracking	A method of treating cancer is provided by implanting one or more brachytherapy spacers or fiducial markers including the matrix material and an anti-cancer therapeutic agent dispersed within the matrix material	[64]
8.	US20190233567A1	Polymethacrylic acid grafted starch	Therapeutics and/or signal molecules	PMAA-g-St-DTPA-Gd nanoparticles could provide different relaxivity at different pH values suggesting their potential use in detection of pH deviations from normal physiological pH 7.4 in tumor tissue or infectious lesions by MR imaging; to deliver therapeutic agent as loaded cargo of the nanoparticles for treatment of any of the following: a neurodegenerative disorder, a neuropsychiatric disorder	[65]
